# Investigation of Presumptive HIV Transmission Associated with Receipt of Platelet-Rich Plasma Microneedling Facials at a Spa Among Former Spa Clients — New Mexico, 2018–2023

**DOI:** 10.15585/mmwr.mm7316a3

**Published:** 2024-04-25

**Authors:** Anna M. Stadelman-Behar, Mika N. Gehre, Liana Atallah, Tegan Clarke, Ana-Alicia Leonso, Francella Jojola, HaoQiang Zheng, Hongwei Jia, Sheryl B. Lyss, William M. Switzer, Scott P. Grytdal, Miranda Durham, N. Mariam Salas, Marla Sievers, Chad Smelser

**Affiliations:** ^1^Epidemic Intelligence Service, CDC; ^2^Epidemiology Response Division, New Mexico Department of Health; ^3^Division of HIV Prevention, National Center for HIV, Viral Hepatitis, STD, and TB Prevention, CDC; ^4^Division of Infectious Diseases, Department of Internal Medicine, University of New Mexico Health Science Center, School of Medicine, Albuquerque, New Mexico; ^5^Public Health Division, New Mexico Department of Health.

**Summary**

**What is already known about this topic?**

Transmission of HIV through cosmetic injection services via contaminated blood has not been previously documented; however, transmission of HIV via unsterile injection practices is a known risk. Determining novel routes of HIV transmission among persons with no known HIV risk factors is important.

**What is added by this report?**

Investigation of multiple HIV infections among persons with no known HIV risk factors who received platelet-rich plasma with microneedling (vampire facials) at an unlicensed New Mexico spa revealed likely HIV transmission associated with these cosmetic injection services.

**What are the implications for public health practice?**

In the absence of known HIV risk factors, clinical and public health staff members might consider cosmetic injection services as a route of HIV transmission. Requiring adequate infection control practices at spa facilities offering cosmetic injection services can help prevent the transmission of HIV and other bloodborne pathogens. Maintenance of client records could facilitate investigations of suspected transmission at such facilities.

## Abstract

HIV transmitted through cosmetic injection services via contaminated blood has not been previously documented. During summer 2018, the New Mexico Department of Health (NMDOH) was notified of a diagnosis of HIV infection in a woman with no known HIV risk factors who reported exposure to needles from cosmetic platelet-rich plasma microneedling facials (vampire facials) received at a spa in spring 2018. An investigation of the spa’s services began in summer 2018, and NMDOH and CDC identified four former spa clients, and one sexual partner of a spa client, all of whom received HIV infection diagnoses during 2018–2023, despite low reported behavioral risks associated with HIV acquisition. Nucleotide sequence analysis revealed highly similar HIV strains among all cases. Although transmission of HIV via unsterile injection practices is a known risk, determining novel routes of HIV transmission among persons with no known HIV risk factors is important. This investigation identified an HIV cluster associated with receipt of cosmetic injection services at an unlicensed facility that did not follow recommended infection control procedures or maintain client records. Requiring adequate infection control practices and maintenance of client records at spa facilities offering cosmetic injection services can help prevent the transmission of HIV and other bloodborne pathogens and ensure adequate traceback and notification in the event of adverse clinical outcomes, respectively.

## Introduction

During summer 2018, the index patient, a woman aged 40–50 years, was evaluated after receiving a positive rapid HIV test result while abroad. Upon evaluation, the patient received a positive HIV antigen/antibody rapid test result, with positive confirmatory results* the same day, indicating stage 1 HIV infection.^†^ The patient reported no injection drug use, recent blood transfusions, or recent sexual contact with anyone other than her current sexual partner, who received a negative HIV test result after the patient’s diagnosis. However, the patient did report exposure to needles during a platelet-rich plasma (PRP) microneedling procedure in spring 2018 at spa A in New Mexico. The procedure involves drawing a client’s blood, separating the blood into its components of plasma and cells, and using single-use disposable or multiuse sterile equipment to inject the PRP into the face for cosmetic purposes, such as skin rejuvenation and reducing the appearance of acne scars (*1*).

## Investigation and Results

NMDOH and CDC investigated cosmetic injection services as a possible transmission route for HIV. The period for active case finding was from spring 2018, when the initial patient received the procedure, to fall 2018 when spa A closed. Spa A’s owner operated without appropriate licenses at multiple locations and did not have an appointment scheduling system that stored client contact information. Investigators compiled and cross-referenced names and telephone numbers from spa A client consent forms, handwritten appointment records, and telephone contacts to create a list of potentially affected clients. The investigative team was not permitted to collect specimens from spa A at the time of the inspection in September 2018, because the inspection was conducted under the purview of the New Mexico Regulation and Licensing Department, which did not have authority to collect specimens. This activity was reviewed by CDC, deemed not research, and was conducted consistent with applicable federal law and CDC policy.^§^

### Identification of Clients at Risk

The investigative team identified 59 clients at risk for exposure, including 20 who received PRP with microneedling at spa A, and 39 who received other injection services (e.g., onabotulinumtoxinA [botox]) during the case-finding period. Investigators cross-referenced the client list with the New Mexico state HIV registry and identified one spa A client who received a diagnosis of HIV in 2012.

During 2018–2023, current and former spa A clients who received new HIV diagnoses were reported to NMDOH from clinical providers throughout the state. During this period, a spa A–related HIV case was defined as a new HIV diagnosis in a patient with previous receipt of blood product or any injection services provided by spa A’s owner^¶^ from 2017 until closure of the unlicensed operation in fall 2018, or who had sexual contact with a person who received such spa services. Cases were included only if an HIV nucleotide sequence demonstrated molecular linkage to other HIV sequences from persons with infections associated with spa A.

### Characteristics of Patients

**FIGURE 1:**
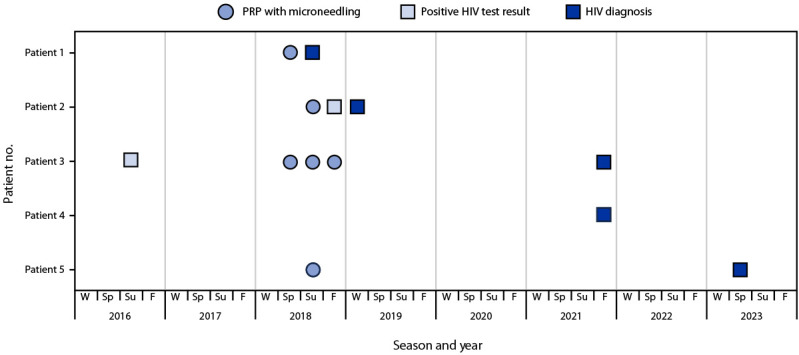
Receipt of platelet-rich plasma and microneedling facial treatments at spa A and HIV screening and diagnosis test results among five patients with HIV infection — New Mexico, 2016–2023 **Abbreviations:** F = fall; PRP = platelet-rich plasma; Sp = spring; Su = summer; W = winter.

By spring 2023, five patients had been identified, including four women and one man who was a sexual partner of one of the four women patients and never received any services from spa A. Blood specimens from the five patients and a former client with a 2012 HIV diagnosis were submitted to CDC for nucleotide sequence analysis to ascertain cluster association and determine case status; all five patients were confirmed to have spa A–related cases. Medical record reviews and clinician interviews were conducted for all confirmed patients. Three patients were interviewed by NMDOH. Patients ranged in age from 40–60 years. HIV diagnoses occurred during summer 2018 through spring 2023 (Figure 1) (Table). Two patients had stage 1 disease, and three had stage 3 disease at the time of diagnosis** (*2*). All four female patients had received PRP with microneedling at spa A.

**TABLE:** Characteristics of patients with confirmed HIV infection associated with receipt of cosmetic injection services at spa A — New Mexico, 2018–2023

Characteristic	Patient no.				
	1	2	3	4*	5
**Sex**	Woman	Woman	Woman	Man	Woman
**Age range, yrs^†^**	40–50	40–50	50–60	40–50	40–50
**Season and year of HIV screening and diagnosis**	Summer 2018	Positive screen: fall 2018; diagnosis: winter 2019	Positive screen: summer 2016; diagnosis: fall 2021	Fall 2021	Spring 2023
**HIV stage^†,§^**	Stage 1	Stage 1	Stage 3	Stage 3	Stage 3
**Spa A services received; season and year received**	PRP with microneedling; spring 2018	PRP with microneedling; summer 2018	PRP with microneedling (multiple procedures); spring and summer 2018	None; NA	PRP with microneedling; summer 2018

**Abbreviations**: NA = not available; PRP = platelet-rich plasma.

* This patient was the sexual partner of a spa A client who received a diagnosis of HIV infection after receiving PRP with microneedling at spa A.

**^†^** At time of HIV diagnosis.

^§^ https://www.cdc.gov/mmwr/pdf/rr/rr6303.pdf

Four of the five patients with confirmed spa A–related HIV infections received at least one PRP with microneedling facial treatment at spa A during May–September 2018. Two of the patients in this cluster (a man and a woman) were engaged in a sexual relationship before and after their diagnoses. Sexual partners of two other patients received negative HIV test results after their partners’ diagnoses, and the remaining patient reported having no sexual partner at the time of diagnosis. Before receiving a diagnosis of confirmed HIV infection, two of the five patients had previously received a positive rapid HIV test result during routine evaluations for life insurance, one in summer 2016, and the other in fall 2018; however, only one patient reported being notified of the positive screening test result and subsequently had their HIV diagnosis confirmed by a primary care provider in winter 2019. The other patient received a confirmed HIV diagnosis after hospitalization with an AIDS-defining illness in fall 2021. One patient received their HIV diagnosis in spring 2023 after hospitalization with an AIDS-defining illness.

The two patients who were engaged in a sexual relationship had stage 3 or chronic HIV infections, indicating that their infections were likely attributed to exposures before receipt of cosmetic injection services. The other three patients in this cluster had no known social contact with one another, and no specific mechanism for transmission among these patients was confirmed. Evidence suggests that contamination from an undetermined source at the spa during spring and summer 2018 resulted in HIV-1 transmission to these three patients.

### Evaluation of HIV Sequences

**FIGURE 2:**
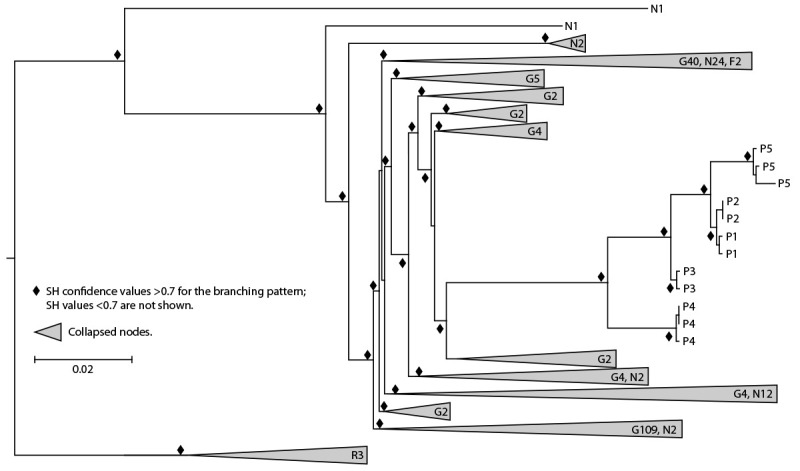
Maximum likelihood phylogeny* of HIV polymerase sequences^†^ from spa A patients 1–5^§^ and client receiving diagnosis of HIV infection in 2012, compared with sequences from GenBank and local HIV surveillance databases — New Mexico, 2018–2023 **Abbreviations:** F = former client; G = genetically related; N = New Mexico controls; P = patients 1–5; R = reference sequence; SH = Shimodaira-Hasegawa. * Collapsed nodes are those with >1 sequence with total number of sequences indicated. Longer branch is associated with higher number of nucleotide substitutions per site. Scale bar for branch length is shown as the number of nucleotide substitutions per site. ^†^ HIV-1 subtype J reference sequences are used as the outgroup. ^§^ Each patient had 2 or 3 sequences included in the analysis.

Whole blood specimens collected from all patients and the former client living with HIV since 2012 were used to generate HIV-1 polymerase (*pol*), *gag*, and envelope (*env*) sequences to evaluate sequence relatedness using established protocols (*3*,*4*). HIV-1 subtype B was determined using the online subtyping tool COMET (*5*). Maximum likelihood phylogenetic analysis was employed to compare the *pol, gag,* and *env* sequences from this investigation with genetically similar sequences from GenBank, the National Institutes of Health genetic sequence database (https://www.ncbi.nlm.nih.gov/genbank), and with 46 local control sequences from routine HIV surveillance in New Mexico in 2018 and 2023 for *pol*. Additional *pol* sequences derived from blood specimens collected by commercial laboratories from three patients within 3 weeks of diagnosis were included in phylogenetic analyses. HIV-1 subtype J sequences were used as an outgroup^††^ for the phylogenetic analyses. Phylogenetic analyses showed that *gag*, *pol*, and *env* sequences from all patients clustered together strongly in a monophyletic clade with high confidence (Figure 2). Sequences from the former client living with HIV, who was not receiving antiretroviral therapy at the time of specimen collection, did not cluster with any New Mexico sequences.

### Investigation of Spa A

In fall 2018, on-site inspection of spa A revealed multiple unsafe infection control practices. A centrifuge, a heating dry bath, and a rack of unlabeled tubes containing blood were located on a kitchen counter. Unlabeled tubes of blood and medical injectables (i.e., botox and lidocaine) were stored in the kitchen refrigerator along with food. Unwrapped syringes were found in drawers, on counters, and discarded in regular trash cans. An autoclave (steam sterilizer) was not found on the premises. Procedure equipment was surface cleaned using ammonium chloride disinfecting spray and benzalkonium chloride disinfecting wipes after each client visit, and disposable electric desiccator tips were cleaned by alcohol immersion and reused.

## Public Health Response

Because Spanish was the first language of many spa A clients, and available client information was limited, NMDOH’s public health response comprised multiple approaches. Direct calls were made to known spa A clients to encourage testing for bloodborne pathogens. Several Health Alert Notifications were sent to providers in New Mexico to ask patients receiving new diagnoses of HIV infection about spa services received before their diagnosis.^§§^ NMDOH communicated the risk for HIV transmission attributed to spa A’s unsterile injection services to the Office of Border Health/Border Infectious Disease Surveillance Group, neighboring jurisdictions through CDC’s Epidemic Information Exchange, and published four press releases during 2018–2023^¶¶^ with information on free testing for current and former spa A clients at state public health offices. NMDOH organized and advertised bloodborne pathogen testing events for current and former spa A clients via social media, radio, newspaper, and television in both English and Spanish. Members of the NMDOH investigative team canvassed community health centers and businesses in predominantly Spanish-speaking neighborhoods to distribute testing information for current and former spa A clients. As a result of these activities, 198 former spa A clients and their sexual partners were tested during 2018–2023. No additional HIV infections were identified, nor were any hepatitis B or hepatitis C infections detected. Free testing remains available for former spa A clients, and the investigation and public health response are continuing.

## Discussion

This investigation is the first to associate HIV transmission with nonsterile cosmetic injection services. A common exposure to spa A among clients without behaviors associated with HIV acquisition helped identify a possible cluster association, and analysis of additional data suggested that HIV transmission likely occurred via receipt of PRP with microneedling facial procedures; however, the source of contamination remains unknown. Although the investigative team was not permitted to collect specimens from spa A, evidence from this investigation supports the likely transmission of HIV through poor infection control practices. This cluster could potentially include additional persons with undiagnosed HIV infection or with a diagnosis of infection but no available sequence for analysis (*3*,*6*).

Incomplete spa client records posed a substantial challenge during this investigation, necessitating a large-scale outreach approach to identify potential cases, as opposed to direct communication with all clients. Requiring maintenance of sufficient client records to ensure adequate traceback by regulated businesses that provide injection services could ensure adequate capability to conduct traceback. NMDOH continues to elicit feedback from former spa A clients to improve future messaging.

### Implications for Public Health Practice

This investigation underscores the importance of determining possible novel sources of HIV transmission among persons with no known HIV risk factors. Requiring adequate infection control practices at spa facilities offering cosmetic injection services can help prevent the transmission of HIV and other bloodborne pathogens. Maintenance of client records could facilitate investigations of suspected transmission at such facilities.

## Footnotes

* Positive HIV-1 and HIV-2 antibody plus HIV-1 p24 antigen test, a negative HIV-1/2 differentiation antibody test, and a detectable HIV-1 RNA qualitative test.

†
https://www.cdc.gov/mmwr/preview/mmwrhtml/rr6303a1.htm?s_cid%20=%20rr6303a1_w

§ 45 C.F.R. part 46, 21 C.F.R. part 56; 42 U.S.C. Sect. 241(d); 5 U.S.C. Sect. 552a; 44 U.S.C. Sect. 3501 et seq.

¶ Or spa A owner’s associates in a location other than the spa.

**
https://www.cdc.gov/mmwr/pdf/rr/rr6303.pdf

†† A more distantly related group that serves as a reference group when determining the evolutionary relationships.

§§
https://www.nmhealth.org/publication/view/general/8339/

¶¶ September 11, 2018: https://www.nmhealth.org/news/alert/2018/9/?view=709; September 21, 2018: https://www.nmhealth.org/news/information/2018/9/?view=712; April 29, 2019: https://www.nmhealth.org/news/alert/2019/4/?view=762; July 5, 2023: https://www.nmhealth.org/news/alert/2023/7/?view=1988
